# Revision of *Kadua* (Rubiaceae) in the Marquesas Islands, French Polynesia, with description of the new species *K. lichtlei*

**DOI:** 10.3897/phytokeys.4.1601

**Published:** 2011-07-12

**Authors:** Warren L. Wagner, David H. Lorence

**Affiliations:** 1 Department of Botany, MRC-166, National Museum of Natural History, Smithsonian Institution, P.O. Box 37012, Washington, DC 20013-7012; 2 National Tropical Botanical Garden, 3530 Papalina Road, Kalaheo, HI 96741 USA

**Keywords:** Conservation, French Polynesia, *Kadua*, Marquesas Islands, Rubiaceae

## Abstract

During the preparation of the Vascular Flora of the Marquesas Islands three new species of *Coprosma* (Rubiaceae, tribe Anthospermeae) have come to light and are described herein: *Coprosma fatuhivaensis* W. L. Wagner & Lorence, *Coprosma meyeri* W. L. Wagner & Lorence, and *Coprosma temetiuensis* W. L. Wagner & Lorence. Descriptions, illustrations, conservation status, and specimen citations are provided. Amended descriptions of three previously described Marquesan *Coprosma* species are also provided as well as a key to the species, four of which fall into the Critically Endangered (CR) and two into the Endangered (EN) category. With the description of these the new species, *Coprosma* becomes the sixth largest lineage in the Marquesas Islands with six species after *Psychotria* (one lineage which has 9 spp.), *Cyrtandra* (8 spp.), *Bidens* (8 spp.), *Melicope* (7 spp.), and *Ixora* (7 spp.).

## Introduction

Until recently the Marquesas Islands were relatively poorly explored botanically. Prior to initiation of the Flore de la Polynésie française project under the auspices of Jacques Florence at IRD (formerly ORSTOM) no species of Rubiaceae belonging to tribe Spermacoceae were known from these islands (Brown 1935). Collecting there intensified greatly with the onset of this project, and Florence and collaborators discovered two distinctive woody species of *Kadua* Cham. & Schltdl.One additional related species was discovered during the collecting phase of the current Vascular Flora of the Marquesas Islands project under the direction of David H. Lorence and Warren L. Wagner (Wagner and Lorence 1997; see website at http://botany.si.edu/pacificislandbiodiversity/marquesasflora/index.htm). These new taxa were published as *Hedyotis lucei*, *Hedyotis nukuhivensis*, and *Hedyotis tahuatensis* by Florence and Lorence (2000). Additional field work in 2003–2004 revealed the presence of yet another species on Ua Huka apparently not closely related to the other three which is described below.

Generic delimitations in *Hedyotis* L. and related genera in Hedyotidinae have not been fully resolved, although recent morphological and molecular studies suggest that *Hedyotis* s. str. is an Old World genus ranging from southeastern Asia to the Caroline Islands of Micronesia and with two widespread species ranging into western Polynesia (Church 2003; Terrell and Robinson 2003; Kårehed et al. 2008; Groeninckx et al. 2009). Virtually all the Polynesian species, the majority Hawaiian, were formerly placed in *Hedyotis* (Fosberg 1943; Wagner et al. 1990). However, results from a recent study of Hawaiian species of *Kadua*, focusing on capsule and seed morphology, revealed that these characters of Hawaiian and certain South Pacific species are distinct from other Asian and western Pacific species of *Hedyotis*, and consequently they have been transferred to the genus *Kadua* (Terrell et al. 2005). *Kadua* now comprises some 30 species including this new species and *Kadua haupuensis* Lorence & W. L. Wagner, a new species recently described from Kaua`i (Lorence et al. 2010).

Based on their salverform, fleshy corollas with appendaged lobes and non-diplophragmous capsules (i.e., that do not separate into paired cocci after dehiscing), the three Marquesan species formerly placed in thegenus *Hedyotis* (Florence & Lorence 2000) have been transferred to the genus *Kadua* by Terrell et al. (2005). These include *Kadua lucei* (Lorence & J. Florence) Lorence & W. L. Wagner, *Kadua nukuhivensis* (J. Florence & Lorence) Lorence & W. L. Wagner, and *Kadua tahuatensis* (Lorence & J. Florence) Lorence & W. L. Wagner.

Results of an unpublished molecular analysis (Motley 2003) place species of the Hawaiian *Kadua* sect. *Kadua* in the same clade as sect. *Protokadua* with a single Hawaiian species, sect. *Gouldiopsis* with four Hawaiian species, and sect. *Austrogouldia* with six species including two Marquesan species (only *Kadua nukuhivensis* and *Kadua tahuatensis* were examined), two from the Society Islands, and *Kadua rapensis* F. Br. from Rapa, as well as the unispecific sect. *Oceanica* with a single species, *Kadua romanzoffiansis* Cham. & Schltdl. from southeastern Polynesia. Together these taxa constitute the large Hawaiian and French Polynesian clade (Motley 2003).

## Methodology

All measurements given herein are taken from dried herbarium specimens, although certain features such as shapes were supplemented with information from alcohol-preserved flowers and fruits, field notes, and digital photos. Measurements are presented in the descriptions as follows: length × width, followed by units of measurement (mm or cm). All specimens cited in this paper have been seen by the authors. Specimens from the following herbaria were studied: AD, BISH, BR, K, MO, NY, P, PAP, PTBG, and US. The area of occupancy (distribution) for this species was calculated using herbarium collection data and field observations, and the conservation status is proposed following the IUCN Red List Category criteria (IUCN 2001; www.iucnredlist.org/info/categories_criteria2001).

## Systematics

### Key to species of *Kadua* in the Marquesas Islands

**Table:** 

1a	Inflorescences with 80–300 flowers; corolla tube 1.8–2.2 mm long; capsules 4–5 mm long, 3.5–4 mm wide; seeds 0.5–0.6 mm long, 0.35–0.4 mm wide, irregularly ovoid to ellipsoid	*Kadua lichtlei*
1b	Inflorescences with 12–30 flowers; corolla tube 13–28 mm long; capsules 7–22 mm long, 6–12 mm wide; seeds 0.9-1.3 mm long, irregularly trigonous or angulate (unknown in *Kadua lucei*)	2
2a	Inflorescences 3–6 cm long; corolla tube 13–16 mm long; Tahuata	*Kadua tahuatensis*
2b	Inflorescences 8–13 cm long; corolla tube 22–28 mm long; Nuku Hiva; Fatu Hiva	3
3a	Corolla lobes 8–10 mm long; capsules 7–8 mm long, 6 mm wide; Fatu Hiva	*Kadua lucei*
3b	Corolla lobes 10–15 mm long; capsules 15–22 mm long, 7–12 mm wide; Nuku Hiva	*Kadua nukuhivensis*

#### Kadua lichtlei

Lorence & W.L.Wagner sp. nov.

urn:lsid:ipni.org:names:77112739-1

http://species-id.net/wiki/Kadua_lichtlei

Figs. 1 2A,B 3 

##### Latin.

Differt a congeneribus Marquesanis laminis late ovatis vel late ellipticis vel rotundis (3–) 5–17.5 × (1.8–) 3.5–11.5 cm, inflorescentia 6.5–14 × 9–12 cm, floribus in inflorescentibus 80–300, parvis, hypanthio 1–1.8 mm longo, corollae tubo 1.8–2.2 mm longo, corollae lobis 1.5–2 mm longis, et capsulis minoribus 4–5 × 3.5–4 mm.

##### Type.

**Marquesas Islands:** Ua Huka: Hane/Hokatu cliff zone, 520m elevation, 14 December 2003, K.R Wood and J.-Y. Meyer 10554 (holotype PTBG-44091!; Isotypes AD!, BISH!, BR!, K!, MO!, NY!, P!, PAP!, US!).

##### Description.

*Shrub or small tree* reaching 4 m tall, glabrous except for inflorescence, moderately branched, branches diffuse or often decumbent, twigs 3–3.5 mm in diam., internodes compressed,bark smooth to striate, dark brown. *Leaves*opposite, those of a pair at a node equal or sometimes unequal, blade (3–) 5–17.5 × (1.8–) 3.5–11.5 cm, broadlyovate, broadly elliptic, broadly obovate-elliptic or subcircular, chartaceous, glabrous, when fresh glossy dark green above, light green beneath, costa greenish white, margins entire, base acute to obtuse or rounded, shortly decurrent, apex obtuse or rounded, tip abruptly short acuminate, 0.5–1.5 cm , secondary veins 6–9(–11) per side, festooned brochidodromous, venation prominulous and conspicuous to 3° adaxially and to 4° abaxially; petioles (0.5–)1–2 cm long, winged distally; stipules interpetiolar (occasionally also intrapetiolar), fused with adaxial petiole bases, the body forming a short, broadly triangular sheath 1.5–3 × 3–7 mm, apiculate or with a short lateral ridge, glabrous, persistent. *Inflorescences* terminal, 6.5–14 × 9–12 cm (including the corollas), cymose-paniculate or -corymbiform, trichotomous, 80–300-flowered, sessile or sometimes with peduncle 3–4 cm long, the basal primary branches subtended by a pair of short petiolate, ovate foliaceous bracts 2–3.5 cm long, branching to the 4° (–5°), axes and pedicels minutely papillose-puberulent, subtended by brown triangular acuminate bracts 0.5–2 × 0.5–1 mm. *Flowers* hermaphroditic, apparently monomorphic and protandrous, borne in dichasial cymules on ultimate branches, central flower often sessile, lateral ones on pedicels to 4 mm long, hypanthium green when fresh, 1–1.8 × 1.2–1.6 mm, broadly obovoid or obconical, tuberculate, calyx tube 0.2–0.4 mm long, glabrous externally and internally, calyx lobes 4 (–5), 0.2–0.5 mm long, triangular, glabrous;corolla in bud 4-angular, apex obtuse, slightly or not depressed, at anthesis shortly hypocrateriform, when fresh with white lobes and green tube, tube 1.8–2.2 × 1–1.3 mm medially, externally and internally glabrous, lobes 4, triangular-ovate, 1.5–2 × 1.3–1.6 mm, apex with a small incurved appendage, externally papillose, internally rugulose; anthers 4, 0.7–1.4 mm long, ellipsoid, apiculate, bilobed basally, sessile, attached below top of tube, tips exserted; style 1.5 mm long, stigma lobes 0.5 mm long, included in tube. *Capsules* 2/3 inferior, broadly obovoid to subglobose, 4–5 × 3.5–4 mm in diam.,apex (beak portion above the calyx) rounded to conical, glabrous, dark brown when fresh, vascular bundles becoming visible with age, loculicidal, apex splitting into 4 segments. *Seeds* c. 200, dull brown, 0.5–0.6 × 0.35–0.4 mm, irregularly ovoid to ellipsoid, laterally compressed, laterally cuneate with a marginal punctiform hilum, the testa irregularly reticulate with areoles enclosing granulate-verrucose mounds.

##### Distribution.

This new species is known only from Ua Huka, Marquesas Islands, where it is apparently restricted to the Hokatu cliff zone above Hane village.

##### Ecology.

Rare and localized, this new species occurs in mixed wet shrubland and herbland growing on basalt cliffs and rock outcrops above wet forest of *Hibiscus tiliaceus* L.*, Pandanus tectorius* Parkinson, and *Freycinetia impavida* (Gaudich. ex Hombr.) B.C. Stone. Other associates include species of *Bidens*, *Boehmeria*, *Maytenus*, *Peperomia*, *Alyxia*, *Morinda*, *Phyllanthus*, *Miscanthus*, *Macropiper*, *Xylosma*, and diverse pteridophytes. It was collected in flower in December and June (in bud), and in fruit in June and December (old fruit with a few seeds).

##### Etymology.

We are pleased to name this new species in honor of Mr. Léon Lichtle, Mayor of Ua Huka, for his generous help and logistic support when we conducted field work on the island and also in recognition of his strong support for conserving the island's native flora and fauna.

##### Conservation status.

The area of occupancy (distribution) for the species was calculated using herbarium collection data and field observations, and the conservation status is proposed following the IUCN Red List Category criteria (IUCN 2001). IUCN Red List Category: **Critically Endangered** (CR): B2a, B2b i-iii; D: B2: total area of occupancy less than 10 km2 (ca. 5 km2). B2a, a single population known; b (i–iii), habitat continuing decline inferred. The suitable habitat for *Kadua lichtlei* on Ua Huka (ca. 83 km2) is indicated as an endangered environment, threatened feral animals and invasive plants, reducing the extent of the forest. Estimated population size is about 30 individuals known only from the type locality (*Wood & Meyer 10514*). Threats include invasion by weeds including *Psidium guajava* L. and *Ageratum conyzoides* L., browsing by goats, and landslides. Several plants are in cultivation at the National Tropical Botanical Garden grown from seed (NTBG accession no. 040036, ex *Wood 10514*).

##### Specimens examined.

**Marquesas Islands:** Ua Huka: Hane valley, ridge and cliffs above tiki marae, back of valley on west side, 8°54.48'S, 138 °3.54'W, 518 m, 12 Jun 2004, Perlman et al. 19007 (BISH, P, PAP, PTBG, US); Hane/Hokatu cliff zone, 520m, 11 December 2003, Wood & Meyer 10514 (BISH, P, PAP, PTBG, US), 550m, 14 December 2003, Wood & Meyer 10544 (PTBG, US), 10547 (PTBG, US), 12 June 2004, 8°54.72'S, 139 °31.60'W, 520 m, Wood et al. 10737 (PTBG), 19738 (PTBG, US).

##### Cultivated.

**Hawaiian Islands:** Kaua`i: Koloa District, National Tropical Botanical Garden, horticulture center nursery, 5 October 2005, *Lorence 9476* (PTBG, US).

##### Discussion.

**Figure 1.**
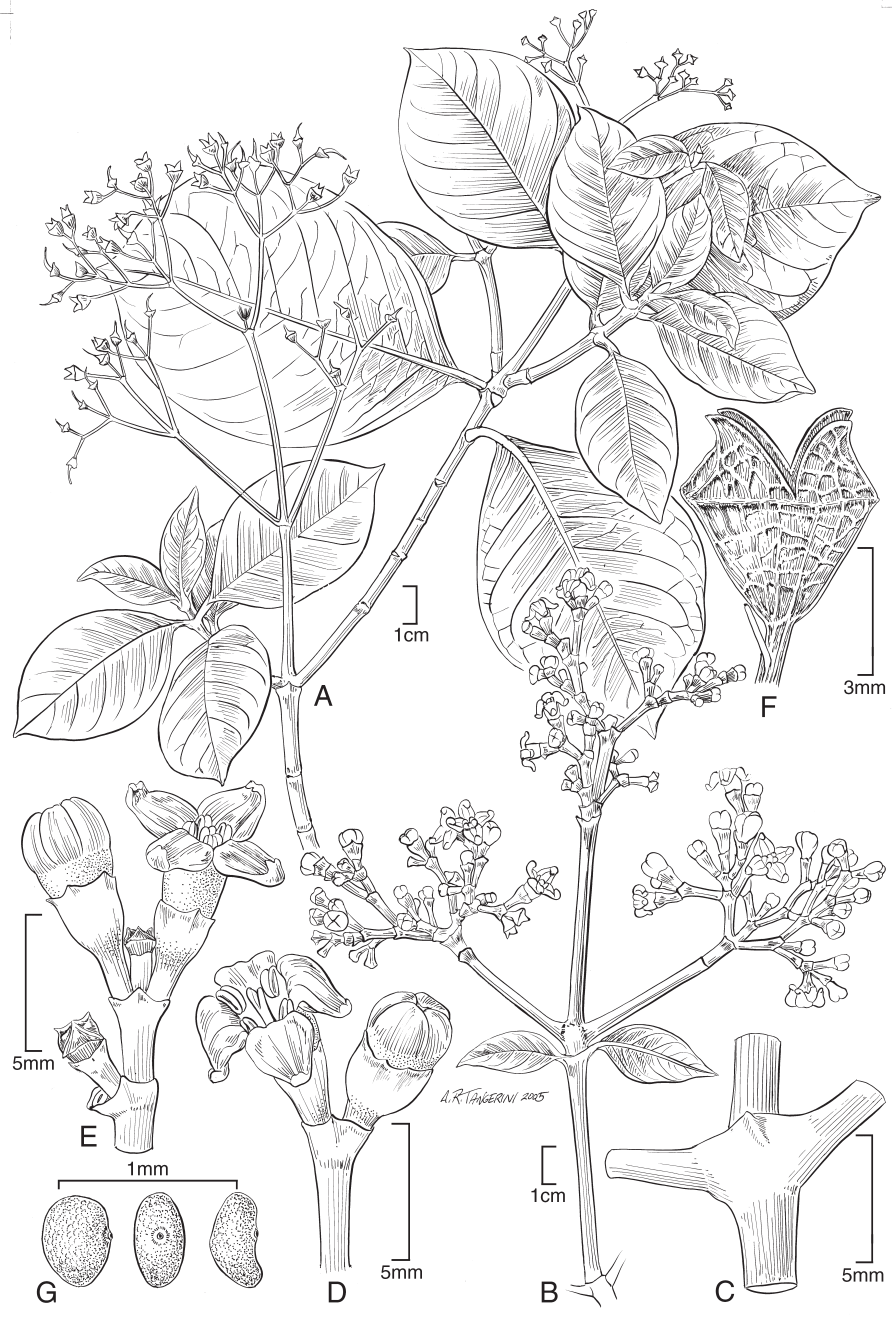
*Kadua lichtlei* Lorence & WL Wagner **A** habit, fruiting branch **B** inflorescence **C** node showing stipule and petiole bases **D,** **E** flowers in bud and at anthesis **F** mature capsule, dehisced **G** seeds, lateral and dorsal (center) views. **A, F, G**. based on the type collection Wood & Meyer 10554; **B, C, D, E** based on Lorence 9476.

Within the Marquesan clade *Kadua lichtlei* differs markedly from the other three species by a number of characters, notably its broadly elliptic to broadly ovate or subcircular leaf blades, much smaller and more numerous flowers (80-300 per inflorescence), and smaller capsules. In addition, seeds of *Kadua nukuhivensis* and *Kadua tahuatensis* (those of *Kadua lucei* were not available) are more sharply angulate with well defined marginal ridges. Seed morphology has proven useful in the infrageneric classification of *Kadua* (Terrell et al. 2005). Seeds of *Kadua lichtlei* are laterally compressed and laterally cuneate with a marginal punctiform hilum, ovoid to ellipsoid, and irregularly angulate with an irregularly reticulate testa with areoles enclosing reticulate-verrucose mounds (Fig. 1G, 2A,B). Its seed morphology suggests an affinity to species of the Hawaiian *Kadua* sect. *Kadua* (Terrell et al. 2005). However, we here place *Kadua lichtlei* in section *Austrogouldia*, partly based on the fact that the intra-areolar seed surface has reticulate verrucose sculpturing and is somewhat different from that in sect. *Kadua*. Seeds of species examined so far in this section (*Kadua nukuhivensis* and *Kadua tahuatensis*) link section *Austrogouldia* to the Hawaiian sect. *Kadua*.

**Figure 2.**
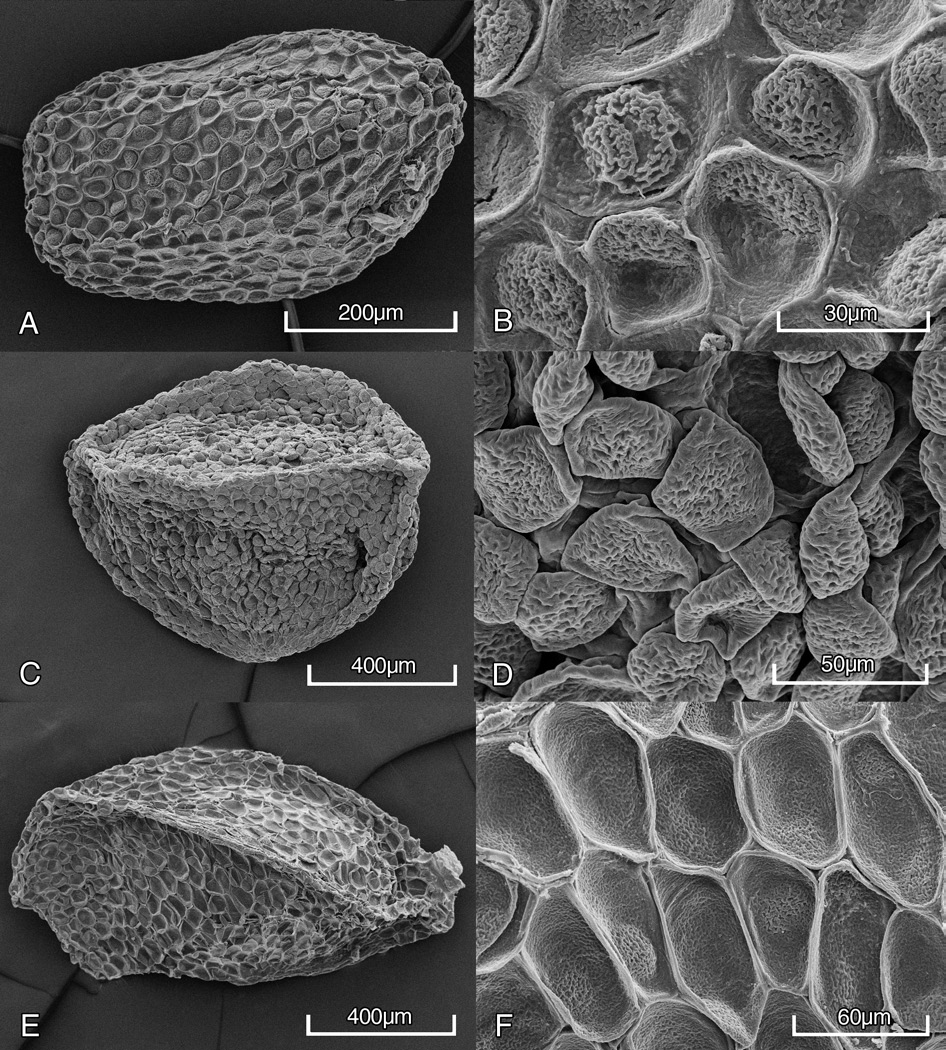
Seeds of three Marquesan *Kadua* species, whole seed and surface detail **A, B** *Kadua lichtlei*, Wood 10514 (PTBG) **C, D** *Kadua nukuhivensis* **C** Perlman 15029 (PTBG) **D** Perlman 15054 (PTBG) **E, F** *Kadua tahuatensis* Perlman 16020 (PTBG).

#### Kadua lucei

(Lorence & J.Florence) W.L.Wagner & Lorence, Syst. Bot. 30 : 832 2005.

http://species-id.net/wiki/Kadua_lucei

Hedyotis lucei Lorence & J. Florence (Adansonia, sér. 3, 22: 225, 2000). [Basionym]

##### Type.

**Marquesas Islands**: Fatu Hiva: au pied de la crête à Touaouoho, env. 1000 m, 3 Aôut1999, J.-P. Luce s.n. (Holotype P!)

##### Description.

*Glabrous shrubs* 1.5–2 m tall, leafy twigs 4–6 mm in diam., internodes compressed, bark brown, smooth to striate or rugulose. *Leaves* of a pair equal to subequal, blade elliptic to obovate-elliptic, (5.3–)10–14.5 × (3.3–)7–10 cm, discolorous, chartaceous to subcoriaceous, base acute to obtuse or rounded, apex acute to obtuse or rounded, often abruptly short acuminate, margin entire, weakly revolute; secondary veins 8–10 pairs, loosely brochidodromous, secondary veins prominulous on both surfaces, tertiary veins slightly prominulous below; petioles purple when fresh, 2–4 × 2–5 mm, stout, adaxially flattened and slightly winged; stipules inter- and intrapetiolar, apex shortly cuspidate, sheath semicircular, 3 × 4–7 mm, fused with the adaxial petiole bases. *Inflorescences*(only seen with old flowers and young fruits) terminal, corymbiform cymose, 8–9 × 6 cm, peduncle 35 mm long, 2.5–3 mm in diameter, compressed, primary axis 2.5–3 cm × 2–2.5 mm with two pair of secondary branches, the lower pair often branching once again, the upper pair unbranched, the ultimate branches each bearing 2–3 flowers, lower bracts foliaceous, suborbicular, 15 mm in diam. *Flowers* glabrous, about 30 per inflorescence, bracts broadly triangular, scarious, 1 × 1.5 mm, pedicels stout, 2–3 × 0.5–1 mm; hypanthium obconical, 2–3 mm long, calyx limb cupuliform, 2–3 mm long, calyx lobes 4, broadly triangular, 0.5–0.7 × 1.5 mm; corolla fleshy, white when fresh, buds not seen, at anthesis salverform, tube purple tinged when fresh, 22–26 mm long, 1.7–2 mm wide medially, lobes 4, linear to oblong, recurved, 8–10 × 1.5–2 mm, apex with a hook-like appendage 0.5–1 mm long; flowers possibly dimorphic; stamens included, anthers linear, 3–3.5 × 0.3–0.4 mm, connective attached 2 mm from apex of tube; style included, 20 mm long, two stigmatic lobes linear, 2.5 mm long. *Fruits* on stout pedicels 5–7 mm long, capsules turbinate to obovoid-turbinate, old capsules 7–8 × 6 mm, consisting of network of persistent vascular bundles enclosing endocarp, apex (beak portion above the calyx) 1–1.5 mm long. Seeds not seen.

##### Distribution.

Marquesas Islands, Fatu Hiva where known only from a single small population on the summit ridge between Tekou and Touaouoho peaks.

##### Ecology.

This new species was collected at 915–1000 m elevation on a steep, precipitous ridge crest in wet shrubland with species of *Alsophila*, *Freycinetia*, and *Histiopteris*.

##### Etymology.

The specific epithet honors its discoverer and first collector, Mr. Jean-Pierre Luce, an amateur naturalist, in recognition of his efforts to explore the most rugged mountainous zones of the Marquesas and thus increase our knowledge of their flora and vegetation.

##### Conservation status.

The suitable habitat for *Kadua lucei* on Fatu Hiva (ca. 85 km2) is indicated as an endangered environment, threatened by feral animals and invasive plants, reducing the extent of the forest. Estimated population size is about 3–4 individuals. Following the criteria and categories of IUCN (2001) *Kadua lucei* is assigned a preliminary Red List status of **Critically Endangered** (CR): B2a, B2b (i–iii); D: B2: total area of occupancy less than 10 km2 (ca. 5 km2). B2a, a single population known; b (i–iii), habitat continuing decline inferred. D, population estimated to number fewer than 250 individuals.

##### Specimens examined.

**Marquesas Islands:** Fatu Hiva: sous la crête entre Tekou et Touaouoho, 915 m, 15 février 2000 (st), J.-Y. Meyer & J.-P. Luce 835 (PAP**,** PTBG).

##### Discussion.

This species is apparently related to *Kadua tahuatensis* from which it differs by its larger inflorescence reaching 8–9 cm long and 6 cm wide, more numerous flowers, about 30 per inflorescence, and larger white corollas with a longer purple-tinged tube 22–26 mm long. Although mature fruits of *Kadua lucei* are not known, old fruits are smaller than those of *Kadua nukuhivensis* and *Kadua tahuatensis*.

#### Kadua nukuhivensis

(Lorence & J. Florence) W.L.Wagner & Lorence, Syst. Bot. 30 : 832, 2005.

http://species-id.net/wiki/Kadua_nukuhivensis

Fig. 2C, D.

Hedyotis nukuhivensis J. Florence & Lorence (Adansonia sér. 3, 22: 224, 2000). [Basionym]

##### Type.

**Marquesas Islands**: Nuku Hiva: route Toovii–Terre Déserte, 5 km après le col, 8°52'S, 140°10'W, 1020 m, 5 juin 1984, J. Florence 6914 (Holotype P!; Isotypes BISH!, P!, PAP!, PTBG!, US!).

##### Description.

*Glabrous shrubs or small trees* 2.5–6 m tall, trunk to 20 cm in diam., leafy twigs cylindrical, 7–9 mm in diam., internodes compressed, bark grayish- to blackish-brown. *Leaves* of a pair equal to sometimes unequal, blade obovate to obovate elliptic or oblong, 8–18 × 6–10.5 cm, chartaceous to subcoriaceous, drying brown or blackish-brown, base cuneate, decurrent, apex obtuse to rounded, secondary veins 7–9 pairs, loosely brochidodromous, prominulous above, tertiary veins slightly prominulous, higher order venation obscure, midrib sulcate above, rounded below, margin entire, plane to slightly revolute; petioles 8–13 × 2–3 mm in diam., sulcate; stipules inter- and intrapetiolar, sheath truncate, cupuliform, 2.5–3 × 5–6 mm, caducous but becoming thickened and horseshoe-like on adaxial surface of petioles. *Inflorescences* terminal and rarely also axillary in upper leaf axils, cymose, corymbiform, 7–13 cm long including corollas, on a stout, compressed peduncle 3–6 cm long or sessile with a pair of basal branches, often subtended by a pair of reduced subsessile leaves, bracts foliaceous, 1.5–3 cm long, caducous. *Flowers* 12–30, possibly dimorphic, fragrant when fresh, subtended by caducous triangular bracts c. 1 mm long, hypanthium obconical, 3 mm long, calyx limb cupuliform, 2 mm long, calyx lobes broadly triangular, 0.3–0.5 mm long; corolla fleshy, white or pinkish white when fresh, apex flat to slightly depressed in bud, at anthesis salverform, tube 26–28 × 2 mm in diam. distally, lobes 4, linear-oblong, recurved, 10–15 × 3–5 mm, apex with a recurved hook-like appendage; stamens with anther tips exserted for 1.5 mm, mucronulate, linear, 3–4 × 1 mm, attached 1.5–2 mm below apex of tube; style 17–19 mm long, included, stigmatic lobes 2, adnate, 3 mm long. *Capsules* on stout pedicels to 6 mm long, turbinate to obpyriform, 15–25 × 7–14 mm, strongly compressed, bisulcate, strongly 8-ribbed, 2/3 inferior, apex (beak portion above the calyx) obtuse, 5–6 mm long, smooth, dehiscence loculicidal then septicidal, old capsules disintegrating leaving network of vascular bundles enclosing the persistent endocarp. *Seeds* 1–1.3 × 0.5–0.9 mm, irregularly angulate to broadly ellipsoid, trigonous, margins thin, testa dull, dark brown, surface finely papillose.

##### Distribution.

Marquesas Islands, Nuku Hiva, where known only from the island's central mountain crest, on the leeward side of the Terre Déserte in the upper Tapuaehu Valley, between 1000 and 1065 m elevation.

##### Ecology.

This species occurs in wet forest with species of *Hernandia*, *Ilex*, *Metrosideros*, and *Weinmannia* in the canopy and the understory with species of *Cyrtandra*, *Melicope*, and *Psychotria*. Numerous pteridophytes occur terrestrially and as epiphytes. *Kadua nukuhivensis* also occurs in shrubland on ridge crests with species of *Alsophila*, *Bidens*, *Dicranopteris*, *Elaphoglossum*, *Freycinetia*, *Pennisetum*, and *Styphelia*.

##### Etymology.

The specific epithet refers to the only known island of occurrence for this species.

##### Conservation status.

The suitable habitat for *Kadua nukuhivensis* on Nuku Hiva (*c.* 340 km2) is indicated as an endangered environment, threatened by human activity (deforestation and fire), feral animals, and invasive plants, thus reducing the extent of the forest. Based on the IUCN criteria and categories this species is assigned a preliminary Red List status of **Endangered** (EN): B1, B2b (i-iii): B1 extent of occurrence <5,000 km2; B2: total area of occupancy less than 500 km2 (c. 75 km2); B2b (i-iii), habitat continuing decline inferred in (i) extent of occurrence, (ii) areas of occupancy, and area, (iii) extent and/or quality of habitat. This status is a revision from VU originally suggested by Florence and Lorence (2000).

##### Specimens examined.

**Marquesas Islands:** Nuku Hiva: Route Toovii—Terre Déserte, haute Tapuaehu, 8°52'S, 140°11'W, 1020 m, 14 fév 1986, Florence 7545 (BISH, P, PAP, US); off the old Airport road west of the summit crest of Peak #1227 m, drainages of Tapueahu Valley, 0.75 miles south of Airport Road, bottom of valley, 3340 ft., 24 sep 1995, Perlman15054 (AD, BISH, MO, P, PAP, PTBG (2), US); Ooumu area (sic), top of Tapueahu off new Hwy, 8°51'53"S, 140°10'63"W, 3500 ft, 23 jun 1997, Wood, Meyer & Luce 6337 (BISH, P, PAP, PTBG, US).

##### Discussion.

*Kadua nukuhivensis* resembles *Kadua tahuatensis*, and molecular evidence places these two as sister species in the same clade within the larger clade of Hawaiian and French Polynesian species (Motley 2003)

**Figure 3.**
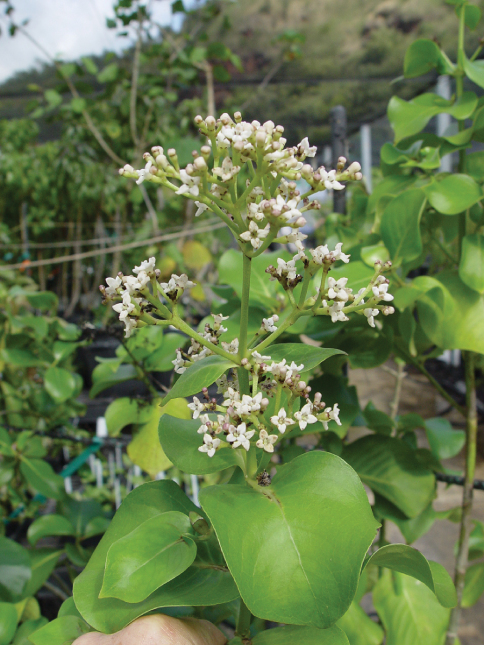
*Kadua lichtlei*, flowering plant growing the National Tropical Botanical Garden nursery, 5 Oct. 2005, Lorence 9476 (PTBG).

#### Kadua tahuatensis

(Lorence & J. Florence) W. L.Wagner & Lorence (Syst. Bot. 30: 832, 2005).

http://species-id.net/wiki/Kadua_tahuatensis

Fig. 2E, F.

Hedyotis tahuatensis Lorence & J. Florence (Adansonia sér. 3, 22: 227. 2000). [Basionym]

##### Type.

**Marquesas Islands**: Tahuata: ridge between Amatea and Haaoiputeomo, south-facing slope, 2580 ft. [780 m] elevation, 19 july 1997, S. P. Perlman 16020 (Holotype: PTBG-30160!; Isotypes: BISH!, MO!,P!, PAP!, US!).

*Glabrous shrubs* to 2 m tall, leafy twigs 4–6 mm in diam., internodes strongly compressed, bark pale brown, smooth to striate. *Leaves* of a pair equal to subequal, blade elliptic to obovate-elliptic, 4.2–15 × 2–8 cm, discolorus, chartaceous to subcoriaceous, base acute to cuneate or narrowly cuneate, apex obtuse to rounded, tip sometimes abruptly short acuminate, secondary veins (5-) 6–9 pairs, weakly brochidodromous, secondary and tertiary veins prominulous on both surfaces, higher order venation obscure, margin thickened, plane; petiole stout, 2–5 × 2–3 mm, adaxially sulcate; stipules inter- and intrapetiolar, sheath cupuliform, truncate, 3 × 5–6 mm, persistent, fused with adaxial petiole surfaces and becoming thickened and horseshoe-like. *Inflorescences* terminal, thyrsiform cymose, 23–25-flowered, 5–6 × 4.5–6 cm (including corollas), on peduncle to 2 cm long, 1.5–2 mm in diam., flattened, primary axis 15 × 1.5–2 mm with 2 pairs of lateral branches, the basal one often branching once, lower bracts foliaceous, ovate, 1.2–2 × 1–1.5 cm, upper branch pair unbranched, ultimate branches ending in 2–3 flowers. *Flowers* glabrous, on stout pedicels 2–3 × 0.8–1.4, compressed, bracts scarious, ovate-trangular, 1 × 1 mm, hypanthium obconical, 3–4 × 1.5–2 mm, calyx limb campanulate, 2–3 × 4–5 mm, lobes ovate-triangular, 1.5-2 × 2-2.5 mm; corolla fleshy, pale green when fresh, lobes with dark purple margins, in bud fusiform with non-depressed apex, at anthesis salverform, tube 13–16 × 1.5–2 mm in diam. medially, lobes 4, linear-oblong, recurved, 8–10 × 1.5–2 mm, apex with a hooked appendage 1 mm long; flowers possibly dimorphic, stamens exserted for 1.5–2 mm, linear, 3-3.5 × 0.5–0.6 mm, attached 1–1.5 mm below apex of tube, apex slightly mucronulate; style included, 11–12 mm long including 2 coalescent stigmatic lobes 2.5 mm long. *Fruits* on stout pedicels 3–8 mm long; capsule tubinate to obovoid-turbinate, 12–20 × 6–8 mm, sub-quadrangular, 2/3 inferior, apex with short beak portion 5–6 mm long above the calyx, dehiscence at first loculicidal then septicidal, old capsules disintegrating into network of vascular bundles enclosing persistent, bisulcate endocarp. *Seeds* irregularly trigonous or angulate, 0.9–1.2 mm long and wide, margins with narrow wing 0.1-0.3 mm wide, testa finely reticulate.

##### Etymology.

The specific epithet refers to the only known island of occurrence for this species.

##### Distribution.

Marquesas Islands, Tahuata, known only from the island's summit crest and high southeastern slopes between 780 and 835 m elevation.

##### Ecology.

Occurs in wet montane forest and shrubland with species of *Alsophila*, *Cheirodendron*, *Crossostylis*, *Hibiscus*, *Metrosideros*, *Reynoldsia*, *Weinmannia*, and pteridophytes including *Asplenium*, *Blechnum*, *Elaphoglossum*, *Lycopodiella* and *Nephrolepis*,

##### Conservation status.

The suitable habitat for *Kadua tahuatensis* on Tahuata (*c.* 61 km2) is indicated as an endangered environment, threatened by feral animals and invasive plants, reducing the extent of the forest. This species is extremely rare, with only five plants known from two localities. Following the criteria and categories of IUCN (2001) it is assigned a preliminary status of **Critically Endangered** (CR): B2a, B2b (i-iii); D: B2: total area of occupancy less than 10 km2 (ca. 5 km2). B2a, a single population known; b (i–iii), habitat continuing decline inferred; D, population estimated to number fewer than 250 individuals.

##### Specimens examined.

**Marquesas Islands:** Tahuata: ridge between Amatea and Haaoiputeomo, southeast-facing slopes over Hanatetena village, 2740 ft. (835 m) elevation, 11 July 1997, Perlman 15954 (P, PAP, PTBG, US).

##### Discussion.

Morphologically *Kadua tahuatensis* closely resembles *Kadua nukuhivensis*, and molecular evidence places these two as sister species in the same clade as *Kadua rapensis* F. Br. and *Kadua romanzoffiensis* Cham. & Schltdl. within the larger clade of Hawaiian and French Polynesian species (Motley 2003).

## Supplementary Material

XML Treatment for Kadua lichtlei

XML Treatment for Kadua lucei

XML Treatment for Kadua nukuhivensis

XML Treatment for Kadua tahuatensis
